# Evaluation of the analgesic efficacies of Dexketoprofen Trometamol and Dexketoprofen Trometamol + Thiocolchicoside combinations in the impacted third molar surgery: Randomised clinical trial

**DOI:** 10.4317/medoral.22590

**Published:** 2018-12-24

**Authors:** Levent Cigerim, Volkan Kaplan

**Affiliations:** 1 DDS, PhD. Assistant Professor, Van Yuzuncu Yil University, Faculty of Dentistry, Department of Oral and Maxillofacial Surgery, Van, TURKEY

## Abstract

**Background:**

Postoperative pain is one of the most common complications. The aim of this study is to evaluate the analgesic efficacies of dexketoprofen trometamol and two different dosages of dexketoprofen trometamol + thiocolchicoside combination in the impacted third molar tooth operation.

**Material and Methods:**

This randomized, double-blind study included 75 patients who did not have any disease. Patients were assigned to 3 groups. Group 1 received 25 mg dexketoprofen trometamol + 4 mg thiocholchicoside, Group 2 received 25 mg dexketoprofen trometamol +8 mg thiocholchicoside, and Group 3 received 25 mg dexketoprofen trometamol. In each group, the analgesic medication was administered twice a day, starting 1 hour before the operation. The level of pain was assessed with VAS.

**Results:**

Patient age varied from 18 to 36 years. Of all patients, 59.2% (n=42) were female and 40.8% (n=29) were male. Drug side effects were observed in 28.17% (n=20) of the patients. Mean 24th hour VAS score was lower in dexketoprofen trometamol + 8 mg thiocolchicoside group compared to dexketoprofen trometamol group (*p*<0.05). There was no statistically significant difference between the three groups regarding drug side effects (*p*>0.05).

**Conclusions:**

Dexketoprofen trometamol + 8 mg thiocolchicoside combination has higher analgesic efficacy compared to dexketoprofen trometamol. More studies are needed to interpret the analgesic and anti-inflammatory effects of thiocholchicoside + dexketoprofen trometamol combination.

** Key words:**Analgesic, dexketoprofen trometamol, thiocolchicoside, third molar surgery.

## Introduction

Lower third molars are the most commonly impacted teeth in jaws (1). Surgical extraction of these teeth is one of the most commonly performed procedures in Oral and Maxillofacial Surgery clinic (2). Most common complications observed after extraction of impacted lower third molar include pain, swelling and trismus (3,4). These complications have negative effect on patient’s return to daily life at the postoperative period and can reduce life quality (5).

Following extraction of impacted lower third molar, the pain starts once the effect of local anesthetic subsides, and is usually worst within the first 24 hours (6). Various methods have been investigated for prevention and/or treatment of postoperative pain. These include medications such as analgesics, steroids, muscle relaxants, antibiotics, as well as methods such as mouthwash use, cold application, low-dose laser therapy, different flap techniques, different closing techniques, drainage, and prp-prf application (7-18). Among these methods, non-steroid anti-inflammatory drugs (NSAID’s) with anti-inflammatory and analgesic effects have been most extensively studied. NSAIDs can be used alone or in combination with paracetamol or opioid analgesics (19,20).

NSAIDs act by inhibiting COX (cyclooxygenase) pathway and prostaglandin synthesis. In addition to the direct effect, inhibition of prostaglandin synthesis also causes an indirect effect by acting on other inflammatory mediators such as kinins (21).

Dexketoprofen trometamol is a propionic acid group of NSAID, and is S (+) enantiomer of ketoprofen. It is a COX-1 and COX-2 inhibitor with well-known anti-inflammatory and analgesic effects. Dexketoprofen trometamol reaches a plasma peak concentration approximately 30 minutes after its oral administration. It has high affinity to plasma proteins (99%) (22).

Thiocholcicoside is a muscle relaxant derived semi-synthetically from the cholchicoside, a natural glycoside. It shows selective agonist action by binding gamma-aminobutyric acid (GABA) receptors and glycinergic receptors. The myorelaxant effect occurs as a results of direct activation of GABA receptors at the spinal level. It also has analgesic and anti-inflammatory effect. The plasma peak concentration is reached approximately 1 hour after oral administration (23).

Studies evaluating the efficacies of NSAID’s in prevention or reduction of pain afer extraction of impacted lower third molar did not yield a consensus on the most effective and safe optimum analgesic, so that there is still research going on this subject. The effervescent tablet form of dexketoprofen trometamol + thiocolchicoside combination is a new drug, and its effect has not been studied. The aim of this study is to evaluate the analgesic efficacies of dexketoprofen trometamol (25 mg dexketoprofen trometamol) and two different dosages of dexketoprofen trometamol + thiocolchicoside combination (25 mg dexketoprofen trometamol + 4 mg thiocolchicoside and 25 mg dexketoprofen trometamol + 8 mg thiocolchicoside) administered 1 hour before extraction of impacted lower third molar.

## Material and Methods

This study was conducted with individuals who came with the indication of extraction of impacted lower third molar upon presenting to Van Yuzuncu Yil University, Faculty of Dentistry, Department of Oral and Maxillofacial Surgery. The inclusion criteria of this randomized double-blinded study were absence of any systemic disease (Class 1 individuals according to American Society of Anesthesiology), absence of smoking/alcohol consumption, absence of allergy to any of the drugs used in the study, absence of pregnancy/lactating state, no history of any medication use during at least 1 week before the operation. Individuals who did not respond despite several telephone calls after the surgery, those who developed alveolitis and received additional treatment for this reason, those who did not show up controls on postoperative days 2 and 7, those who did not fill VAS (visual analogue scale) or filled it incompletely, or those whose operation took more than 30 minutes were excluded from the study.

Presence of impacted lower third molar was confirmed in individuals presenting to the clinic by clinical and radiographic examination. Impacted lower third molar that were Class 1 (Class A or B) or Class 2 according to Pell and Gregory Classification, vertical or mesioangular according to Winter Classiffication, and showed partial or compete bone retention were selected for the study. The individuals were informed about the study, and those who volunteered signed a consent form. The study was approved by Van Yuzuncu Yil University Clinical Research Ethics Committee, and all surgical procedures were carried out in accordance with the Helsinki Declaration.

A total of 75 patients were enrolled in the study. Each patient had one impacted lower third molar with indication of extraction. The medication given to patients were encoded with the numbers 1, 2, 3 by the assistant personnel, and were bottled in different vials. Randomization of medicines was achieved using an online software (http://graphpad.com/quickcalcs/randomize1.cfm). One hour before the operation, the assistant personnel recorded the code of the medicines on the patient file that was enclosed in an envelope, and the envelopes were opened by the same personnel only.

Anesthesia of inferior alveolar nerve, lingual nerve and buccal nerve was achieved using 2 ml ampoules containing 40 mg/ml articaine hydrochloride + 0.01 mg/ml epinephrine (Maxicaine Fort, Vem Pharmaceutical Industry and Trade Co. Ltd, Istanbul, Turkey). 1.2 ml of the solution was deposited for inferior alveolar nerve + lingual nerve, and 0.5 ml of the solution was deposited for buccal nerve. All surgical procedures were performed by the same surgeon following the same surgical procedure. Following elevation of a three-cornered flap, the bone was elevated under physiological cooling with sterile saline, and the teeth were removed with incisions when necessary. The extraction socket was irrigated with sterile saline, and the incision line was closed primarily with 3/0 silk suture. During the postoperative 5 days, patients in Group 1 received 25 mg dexketoprofen trometamol + 4 mg thiocolchicoside effervescent tablet (Dexplus 25/4, Vitalis Pharmaceuticals Industry and Trade Co. Ltd. Istanbul, Turkey) twice daily; patients in Group 2 received 25 mg dexketoprofen trometamol + 8 mg thiocholchicoside effervescent tablet (Dexplus 25/8, Vitalis Pharmaceuticals Industry and Trade Co. Ltd. Istanbul, Turkey) twice daily; and patients in Group 3 received 25 mg dexketoprofen trometamol tablets (Arveles, Ufsa Pharmaceutical Industry and Trade Co. Ltd. Istanbul) twice daily. The patients were advised to use paracetamol (Parol 500 mg tablet, Atabay Pharmaceuticals, Istanbul) only when they needed additional analgesic. In addition to the study medication, mouthwash containing 0.06% chlorhexidine glucanate (Mirafluor chx 100 ml, Hager & Werken GmbH & Co, KG, Duisburg, Germany) was prescribed three times a day. Patients were recommended not to drive a vehicle, etc.

Post-operative controls and measurements were performed by another surgeon who was blinded to treatment allocations. The measurements and other information were recorded in the patient file by the assistant personnel. Wound healing was assessed by evaluation of factors such as wound gap and its size, dry socket, and pus drainage. Drug side effects were questioned at the postoperative controls, and was recorded to patient’s file when present.

Postoperative pain was assessed with VAS at 1st, 2nd, 3rd, 6th, 8th and 24th postoperative hours and 2nd, 3rd and 5th days.

-Statistical Analysis

Data were analyzed with SPSS statistical software (IBM Statistics (21.0). For evaluating study data, in addition to calculating descriptive statistics (mean, standard deviation, median, frequency, proportion, minimum, maximum), comparison of quantitative data between three or more groups was made with one-way ANOVA test for normally distributed data, or Kruskal Wallis test for non-normally distributed data. Mann-Whitney U tests were used in cases which within group differences were detected with Kruskal Wallis test, and *p*<0.05 was accepted as statistically significant.

Sample size was estimated using data from Eroglu and colleagues (24). It was considered necessary to include 20 patients per group to be able to detect a 50% difference in pain levels (type I error frequency: 0.05, power: 0.80). We included 25 patients per group considering losses to the follow up.

## Results

Of 75 patients operated, 4 developed alveolitis and these patients were excluded from the study. Of 71 patients included in the analysis, 59.2% (n=42) were female and 40.8% (n=33) were male. Patients’ age varied from 18 to 36 years, with a mean age of 23.5±3.8 years. Drug side effects were observed in 28.17% (n=20) of the patients. Among patients developing drug side effects, 9.86% (n=7) had stomach complaint, 5.63% (n=4) had headache/dizziness, 1.41% (n=1) had earache, 1.41% (n=1) had stomach complaint + headache/dizziness + palpitation + chills, 9.86% (n=7) had stomach complaint + headache/dizziness ( Table 1).

**Table 1:**
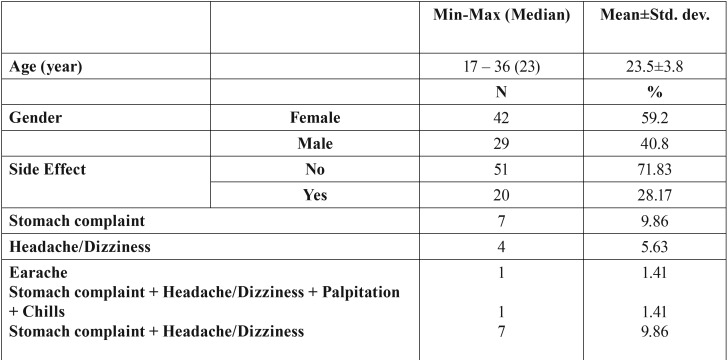
Demographic characteristics and descriptive statistics.

Patients’ age and gender distributions and side effect rates did not show statistically significant differences according to drug groups (*p*>0.05) ( Table 2).

**Table 2:**
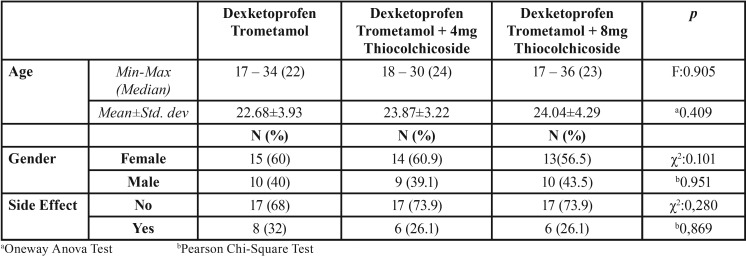
Evaluation of age, gender and side effects according to drug groups.

**Figure 1:**
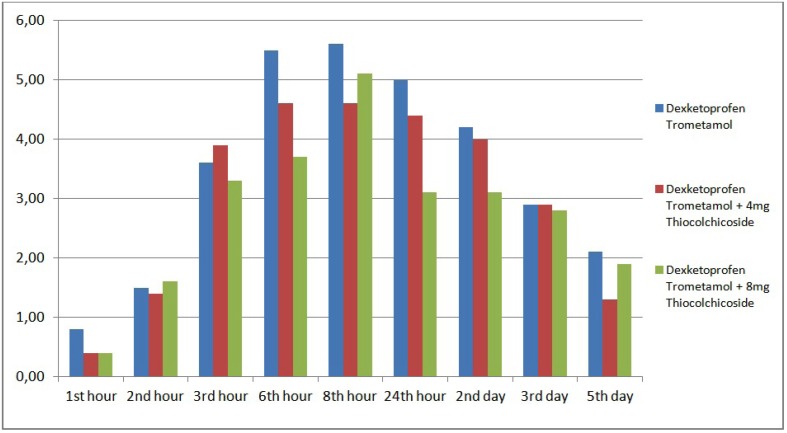
The distrubition of VAS scores during assessment times among the groups.

Patients’ mean VAS scores at 1st, 2nd, 3rd, 6th and 8th hours and 2nd, 3rd and 5th days did not show statistically significant differences according to drug groups, however, mean VAS scores of patients in dexketoprofen trometamol + 8 mg thiocolchicoside were lower than mean VAS scores of patients in dexketoprofen group at all times (*p*>0.05). Mean 24th hour VAS scores of patients in dexketoprofen trometamol group was significantly higher compared to that of patients in dexketoprofen trometamol + 8 mg thiocolchicoside (*p*<0.05). The difference in 24th hour VAS scores between dexketoprofen trometamol + 4 mg thiocolchicoside and dexketoprofen trometamol + 8 mg thiocolchicoside groups, or between dexketoprofen trometamol + 4 mg thiocolchicoside and dexketoprofen trometamol groups was not statistically significant (*p*>0.05) ( Table 3, Fig. 1).

**Table 3:**
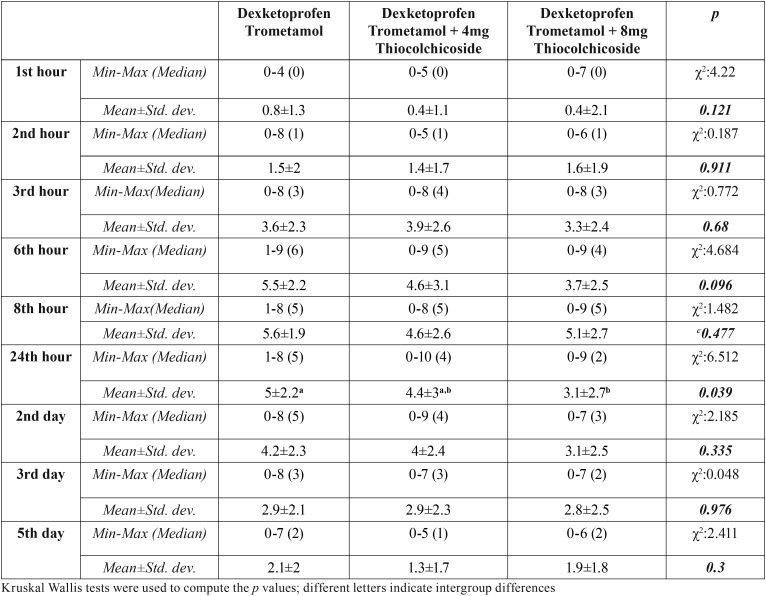
Evaluation of the VAS scores according to drug groups.

Highest mean VAS scores was observed at the 8th hour in all 3 drug groups. Mean VAS scores in dexketoprofen trometamol and dexketoprofen trometamol + 4 mg thiocolchicoside groups started to fall on the 3rd day, whereas in dexketoprofen trometamol + 8 mg thiocolchicoside group, they started to fall at the 24th hour.

**Figure 2:**
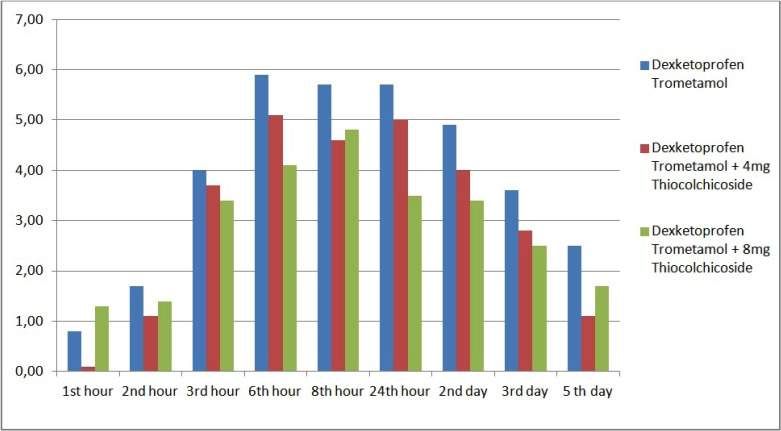
The distrubition of VAS scores during assessment times among the groups in female patients.

**Figure 3:**
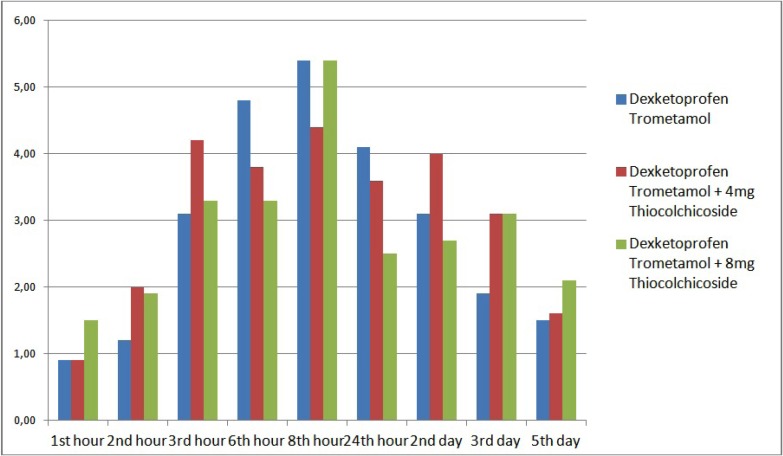
The distrubition of VAS scores during assessment times among the groups in male patients.

Comparing of mean VAS scores among female patients, no significant differences were observed according to drug groups (*p*>0.05) ( Table 4, Fig. 2). Comparing of mean VAS scores among male patients, no significant differences were observed according to drug groups (*p*>0.05) ( Table 4, Fig. 3).

**Table 4:**
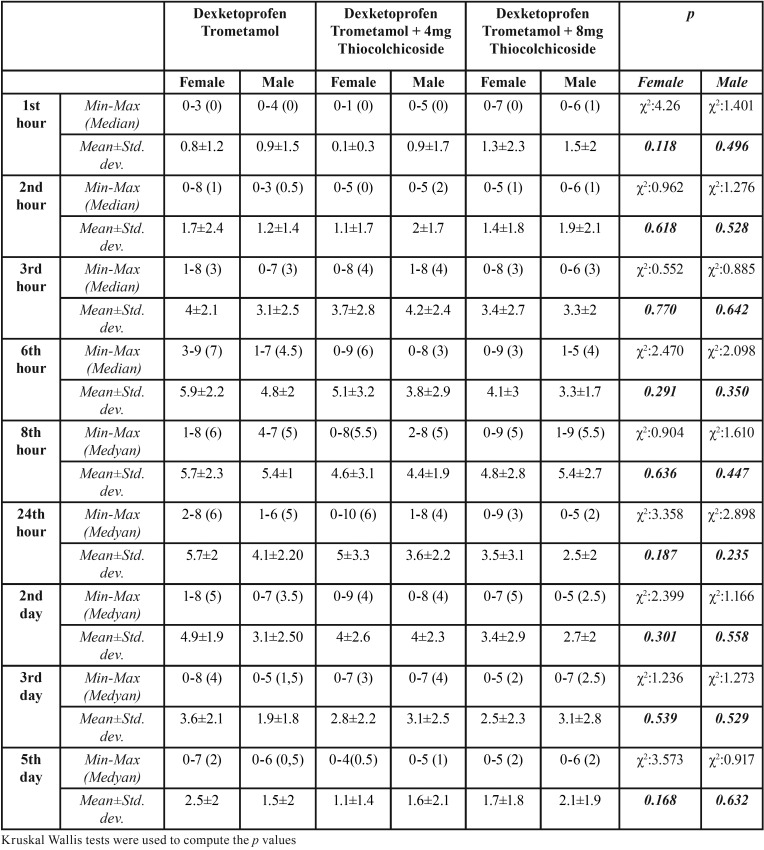
Evaluation of the VAS scores according to drug groups in female and male patients.

## Discussion

One of the most common complications observed after extraction of impacted lower third molar is pain. There are many studies related to management and treatment of pain (25). Since NSAID’s create analgesia due to their anti-inflammatory effects, and they have relatively less side effecs, they have been frequently used and investigated in studies related with pain, and they are effective in mild to moderate pain. Paracetamol is a central acting analgesic with little anti-inflammatory effect, and it is effective in mild pain. As it is a safe drug with little anti-inflammatory effect, it has often been used studies for rescue analgesia (7). Although steroids have greater anti-inflammatory effects compared to NSAID’s, they are not desired for long-term use due to their dose-dependent side effects (8). Opiods have no anti-inflammatory effect, the analgesic effect occurs through central action. They have limited use due to their potential for addiction (19). These reasons explain why NSAID’s are preferred in studies on impacted third molar; however, NSAID’s may fail to control severe pain within the first 24 hours, particularly starting after the 6th postoperative hour. It is thought that combination of NSAID’s with different ancillary analgesics without increasing its dose can create synergistic analgesic effect, and thus potential side effects can be reduced to minimum while controlling severe pain (20).

In the literature, the analgesic efficacy of dexketoprofen trometamol in extraction of impacted lower third molar has been evaluated in many studies. Bagan and colleagues found dexketoprofen trometamol in 12.5 mg and 25 mg dosages were more effective than 575 mg dipyrone (26). Jackson and colleagues found that analgesic efficacy of 25 mg dexketoprofen trometamol was similar to 50 mg refocoxib, and superior to placebo (27). In the study by Çağıran and colleagues preoperative intravenous 50 mg dexketoprofen trometamol was found to be more effective than placebo (28). Eroglu and colleagues reported that 12.5 mg dexketoprofen trometamol had similar analgesic effect with 500 mg paracetamol (24).

In studies using dexketoprofen trometamol in extraction of impacted lower third molar, the pain was at maximum level at the 4th hour in the study by Çağıran and colleagues, and at 8th hour in the study by Eroglu and colleagues (24,28). Consistent with the literature, we found that pain was maximum at the 8th hour in all three groups in the present study. Pain levels started to decrease by 3rd day in dexketoprofen trometamol and dexketoprofen trometamol + 4 mg thiocolchicoside groups, and by 24th hour in dexketoprofen trometamol + 8 mg thiocolchicoside group. The reason why pain levels started to fall earlier in dexeketoprofen + 8 mg thiocolchicoside group compared to the other two groups is though to be that the effect was more pronounced after receiving the second dose.

In their study, Kirmeier and colleagues investigated the efficacy of tizanidine, a central acting muscle relaxant, in extraction of impacted lower third molar. They administered tizanidine in combination with dexibuprofen, which is a NSAID. 4 mg tizanidine was administered orally at nighttime on the day of surgery and postoperative day 1. They found no difference between the groups that did or did not receive tizanidine regarding mean VAS scores on postoperative 1st, 3rd and 7th days and mouth opening. They observed maximum level of pain at the postoperative 24th hour (29).

In a study by de Santana Santos and colleagues, the analgesic effect of cyclobenzaprine, a centrally acting muscle relaxant, which was started 1 day before surgery and continued on the day of surgery and on the first postoperative day, was compared with the placebo in patients with bilaterally impacted third molar. In addition to cyclobenzaprine, 8 mg dexamethasone and 1 g amoxicillin were administered orally 1 hour prior to surgery. Postoperatively, patients used 750 mg acetaminophen when needed (maximum 4 times a day). No difference was observed between the groups regarding analgesic efficacy at postoperative 4th, 6th, 12th, 24th and 48th hours. The groups did not show difference regarding edema and trismums on postoperative 2nd and 7th days. Maximum pain was observed at postoperative 4th and 6th hours (30).

In this study, the analgesic effect of dexketoprofen trometamol + 8 mg thiocolchicoside was found to be higher than dexketoprofen trometamol at 24th hour. Dexketoprofen + 4 mg thiocolchicoside, showed similar analgesic effect with dexketoprofen trometamol. The analgesic effect of thiocolchicoside increased with increasing dose. The thiocolchicoside used in this study is a central acting muscle relaxant. Other centrally acting muscle relaxants used in the studies, cyclobenzaprine and tizanidine, do not create additional analgesic effect, and this can be explained by the fact that they lack anti-inflammatory action (30). In the study by Kirmeier colleagues tizanidine was started at night before the operation, and this may be the reason for the delayed analgesic effect, and for the lack of efficacy within the 24 hours when the pain is most intense. Additionally, the time intervals for assessment of pain could also have influenced the results. Unlike the other two muscle relaxants (cyclobenzaprine and tizanidine), thiocholchicoside that is used in this study has anti-inflammatory property. It is thought that increased analgesic efficacy of thiocholchicoside at inreasing doses may be related to the anti-inflammatory mechanism of action (29).

When we compare drug side effects observed in studies using dexketoprofen trometamol, the side effects were observed in rates of 10.24% in the study by Bagan and colleagues, 11.9% in the study by Jackson and colleagues, and 0% in the study by Eroglu and colleagues (24,26,27). In the study by De Santana Santos and colleagues, in which cyclobenzaprine was combined with paracetamol, the rate of side effects was reported as 6% (30). In the study by Kirmeier and colleagues, which was the only study that combined a NSAID (dexibrufen) with a muscle relaxant, no drug related side effect was mentioned (29). Unlike the other studies in the literature, drug side effects were observed in 28.17% of the patients in the present study. Most commonly observed side effects were gastrointestinal system disturbances and headache or dizziness. It was expected that thiocolchicoside groups that received this drug for 5 days would have greater rate of side effects, however, similar drug side effects were observed in all drug groups. Drug side effects are thought to be related to dexketoprofen trometamol, which is the common drug in all three groups.

In conclusion, this study was the first study to use the combination of dexketoprofen trometamol + thiocolchicoside and to evaluate their analgesic effect in the extraction of impacted lower third molar. Within the limitations of the study, dexketoprofen trometamol + 8 mg thiocolchicoside was found to be more effective on pain than dexketoprofen trometamol alone at 24th hour. More studies are needed to better interpret the analgesic and anti-inflammatory effects of the thiocolchicoside + dexketoprofen trometamol combination.
